# Seizure prediction in cerebral venous thrombosis– a retrospective single-centre observational study

**DOI:** 10.1007/s10072-025-08256-x

**Published:** 2025-05-28

**Authors:** Patrícia Faustino, Diana Melancia

**Affiliations:** 1 Neurology Department,Hospital de São José, Unidade Local de Saúde São José, R. José António Serrano, 1150-199 Lisboa, Portugal; 2 Cerebrovascular Unit,Hospital de São José, Unidade Local de Saúde São José, Lisboa, Portugal

**Keywords:** Cerebral venous thrombosis, Acute symptomatic seizures, Late seizures, Predictors

## Abstract

Cerebral venous thrombosis (CVT) accounts for 0.5-1% of all strokes and 24–50% of these patients develop acute symptomatic seizures (AS). Clinical and radiological characteristics have been associated with an increased risk of AS in CVT. We aimed to identify clinical and imaging predictors associated with a higher risk of AS in CVT patients.

We conducted a single-centre, retrospective cohort study and included all patients with CVT admitted to our stroke unit between January/2011-December/2022. Our primary outcome was AS occurence. Clinical and radiological characteristics were compared through a logistic binary regression, followed by a multivariable analysis.

We included 156 patients, 80.8% female and a mean age of 41.5 ± 15.2 years. Fifty-two patients (33.3%) had a seizure during follow-up, the majority as AS (30.1%). We found an increased risk of AS in patients with focal signs at presentation (OR 5.35), superior longitudinal sinus (SLS) or cortical vein involvement (OR 5.03; OR 3.94), hemorrhagic lesions or oedema (OR 3.88; OR 4.17) and lesions located in the frontal or the parietal lobe (OR 4.61; OR 4.61). A multivariable analysis was also conducted and only SLS involvement (OR 6.06), cortical vein involvement (OR 2.76) and hemorrhagic lesion (OR 3.47) remained statistically significant.

Seizures occurred in about a third of our CVT patients, the majority as AS. Haemorrhagic lesions, SLS and cortical vein involvement had a stronger association with AS that may raise our awareness for the risk of seizures in this population during the acute phase.

## Introduction

Cerebral venous thrombosis (CVT) accounts for 0.5-1% of all strokes [1] and 24–50% of these patients develop acute symptomatic seizures (AS) [2]. Some clinical and radiological characteristics have been associated in previous studies with an increased risk of AS in CVT, namely pregnancy or puerperium, presentation with focal neurological deficits, a Glasgow Coma Score (GCS) ≤ 8, supratentorial parenchymal lesions with cerebral oedema/infarct (particularly in the frontal lobe), haemorrhagic lesions, sulcal subarachnoid haemorrhage (SAH) and involvement of the superior longitudinal sinus (SLS) and cortical veins [1–6]. There was also an association with encephalopathy and V Leiden factor mutation in a recent observational study [4]. Late seizures (LS) have been defined as occurring after more than seven days after the diagnosis of CVT and occur in approximately 10% of patients in a two-year follow-up, with a high chance of recurrence [7]. Status epilepticus in the acute phase, acute symptomatic seizures, haemorrhagic lesion, subdural hematoma, and decompressive hemicraniectomy have been described as LS predictors [7], and there was also an association with younger age and hormonal contraceptives [4]. 

We aimed to identify clinical and imaging predictors associated with a higher risk of acute and late seizures in a population of CVT patients in a tertiary centre.

## Materials and methods

We conducted a single-centre, retrospective cohort study and included all patients with CVT admitted to our stroke unit between January 2011 and December 2022. Patients with clinical records with more than 50% missing data were excluded.

Baseline variables were collected from patients’ records, including age and gender; in the female patients we also assessed the use of contraceptive drugs or if it happened during pregnancy or puerperium. We also analysed clinical and radiological data at presentation– presentation with focal signs, headache or GCS ≤ 8, cerebral venous thrombosis location (SLS and cortical vein) and parenchymal lesions, hemorrhage, parenchymal oedema and sulcal subarachnoid hemorrhage. For CVT diagnosis, computed tomography (CT) with venography and magnetic resonance (MR) venography was used. Data was also collected when available regarding anti-seizure medication.

We defined our primary outcome as seizure occurrence and subdivided in the presence of acute symptomatic seizures, defined as in between symptom onset and 7 days after diagnosis of CVT [2], and late-onset seizures, defined as occurring after seven days after a diagnosis of CVT [7].

Qualitative variables are presented in percentages and absolute number. Quantitative variables were summarized by mean value and standard deviation or median and interquartile range. Comparisons between patients with and without seizures were conducted, as well as analysis between the subgroups of patients with AS and with LS when possible.

Comparisons between groups were performed through chi-square, t-student or Mann-Whitney test. We compared clinical and radiological characteristics in both groups through a logistic binary regression, followed by a multivariable logistic regression analysis, and presented the results as Odds Ratio (OR). The p value was considered significant if inferior to 0.05. The statistical analysis was conducted using SPSS v25.

Patient data were analysed without identity-revealing elements, and there was exemption for consent attending to the study design.

## Results

We included 156 patients with a mean age of 41.5 ± 15.2 years; 80.8% were female (*n* = 126), with 61.9% under hormonal birth control and 5.6% in the pregnancy/puerperium period. The baseline characteristics of both groups are presented in Table 1.

**Table 1:** Baseline, clinical and radiological characteristics comparison between the two groups

	With Seizures (*n* = 52)	Without Seizures (*n* = 104)	*p* value
Age, years– mean (SD)	42.2 (16.3)	41.1 (14.7)	0.930
Gender, female– n (%)	44 (84.6)	82 (78.8)	0.520
Contraceptive use– n (%)	27 (51.9)	51 (49.0)	1.000
Pregnancy/Puerperium– n (%)	3 (5.8)	4 (3.8)	0.690
Headache at Presentation– n (%)	38 (73.1)	99 (95.2)	**< 0.001**
Focal Signs at Presentation– n (%)	33 (63.5)	27 (26.0)	**< 0.001**
GCS ≤ 8– n (%)	8 (15.4)	11 (10.6)	0.439
CVT SLS– n (%)	37 (71.2)	38 (36.5)	**< 0.001**
CVT cortical vein– n (%)	20 (38.5)	15 (14.4)	**0.001**
Parenchymal lesion– n (%)	44 (84.6)	41 (39.4)	**< 0.001**
Hemorrhagic lesion– n (%)	33 (63.5)	28 (26.9)	**< 0.001**
OEdema– n (%)	25 (48.1)	16 (15.4)	**< 0.001**
Sulcal subarachnoid hemorrhage– n (%)	14 (26.9)	15 (14.4)	0.080

The presenting symptoms at diagnosis were mainly headaches in 87.8% of patients (*n* = 137), focal deficits in 38.5% (*n* = 60) and AS in 30.1% (*n* = 47). Considering the radiological findings, about half of the patients admitted presented with involvement of the SLS (48.1%) and only 22.4% had cortical vein involvement; 45.5% of patients had evidence of a parenchymal lesion, with the most frequent topography being the temporal lobe (*n* = 42, 49.4%). As expected, most lesions had evidence of hemorrhage (*n* = 61, 71.8%) and about half the patients had parenchymal oedema (*n* = 41, 48.2%). Additionally, we found 29 patients (18.6%) with sulcal subarachnoid hemorrhage.

The median follow-up was 2.0 years (IQR 1.5); fifty-two patients (33.3%) had a seizure during follow-up, with the vast majority occurring in the first seven days as AS (*n* = 47, 30.1%) and 10 patients (6.4%) with LS.

There were 40 (25.6%) patients that were started on anti-seizure medication. Unfortunately, it was not possible to ascertain the exact criteria for anti-seizure medication initiation, being according to the decision of the responsible attending.

We found an increased risk of AS in patients with focal signs at presentation (OR 5.35, CI 95% 2.56–11.18, *p* < 0.001), with SLS or cortical vein involvement (respectively, OR 5.03, CI 95% 2.35–10.79, *p* < 0.001; OR 3.94, CI 95% 1.80–8.67, *p* = 0.01), with the presence of hemorrhagic lesions or oedema (respectively, OR 3.88, CI 95% 1.89–7.95, *p* < 0.001; OR 4.17, CI 95% 1.96–8.89, *p* < 0.001) and with lesions located in the frontal or the parietal lobe (respectively OR 4.61, CI 95% 2.05–10.34, *p* < 0.001; OR 4.61, CI 95% 2.05–10.34, *p* < 0.001). We did not find association with gender, hormonal birth control, pregnancy/puerperium period, GCS ≤ 8 (OR 1.83, CI 95% 0.68–4.89, *p* = 0.23), subarachnoid hemorrhage (OR 2.22, CI 95% 0.97–5.10, *p* = 0.06) or temporal lesions (OR 1.90, CI 95% 0.91–4.01, *p* = 0.09).

A multivariable analysis was also conducted and only SLS involvement (OR 6.06, CI 95% 2.11–17.40, *p* = 0.001), cortical vein involvement (OR 2.76; CI 95% 1.08–7.02, *p* = 0.03) and hemorrhagic lesion (OR 3.47, CI 95% 1.27–9.49, *p* = 0.02) remained statistically significant.

Regarding LS occurrence, only 10 (6.4%) patients presented with LS and of these patients five had also presented with AS and all five were started on anti-seizure medication. There were eight patients in which we could not ascertain LS occurrence.

## Discussion

Seizures occurred in about a third of our CVT patients, with the vast majority being acute symptomatic seizures. We identified the involvement of SLS or cortical veins (which might occur in association with distal sinus thrombosis), haemorrhagic lesions, oedema, parietal or frontal lobe involvement, and focal neurological deficits upon presentation as independent predictors for AS, increasing its risk between four to five-fold. Haemorrhagic lesions, SLS and cortical vein involvement had a stronger association with AS that may raise our awareness for the risk of seizures in this population during the acute phase. We also found that parietal lesions increased the risk of AS in our population, which had not been previously described. We understand that there is some overlap between some of these features, namely considering the presence of focal neurological deficits and parenchymal lesions, nevertheless we wanted to highlight both the clinical and radiological features associated with the seizure risk.

Our data regarding LS occurrence and seizures after six months might be underestimated due to the low number of patients with LS in our cohort, and therefore we were unable to perform statistical analysis of significance in this group.

Considering the high epileptogenic potential of haemorrhagic lesions/haemoglobin components that has been previously described [8] there is a theoretical basis for this strong association. This is also true for SLS involvement which leads to poor venous outflow which may aggravate cortical oedema and increase intracranial pressure, being therefore associated with epileptogenic risk [9]. This would raise the question if whether patients that presented with some of the characteristics that have been clearly associated with AS in the literature would potentially benefit from prophylactic seizure medication for a period of time in which their seizure susceptibility is higher. There was a recent publication that proposed a comprehensive nomogram for early seizure prediction [1] which included five factors - focal neurological deficits, a GCS score ≤ 8, haemorrhagic lesions and involvement of the SLS and the frontal lobe, with GCS ≤ 8 having the highest association. The authors postulated that it would allow determination of early seizure probability with a combination of variates that would facilitate decision-making.

The fact that this was an observational study means we could not control the variabilities in the anti-seizure medication and there are also difficulties in the assessment of exact dosages, treatment duration or other confounding factors, as well as since it was conducted in a tertiary centre some patients returned to other hospitals with a more difficult assess to the clinical records and therefore follow-up information.

Additional limitations of our study include the single-centre and retrospective design with consequently higher chance of selection and information bias. The fact that the included patients were admitted to a stroke unit may be responsible for a higher selection bias in our sample, since there is a higher chance that these patients presented with higher disease severity and required monitoring in a highly specialised unit, which may overestimate the proportion of patients that presented with seizures.

All-in-all, our findings were concordant with the literature available and support the need to conduct larger studies, with a strict follow-up and control over the anti-seizure medication provided, since further evidence is needed to determine which patients would potentially benefit from prophylactic anti-convulsive therapy and whether this would impact long-term outcomes.
